# IGSMNet: Ingredient-Guided Semantic Modeling Network for Food Nutrition Estimation

**DOI:** 10.3390/foods14213697

**Published:** 2025-10-30

**Authors:** Donglin Zhang, Weixiang Shi, Boyuan Ma, Weiqing Min, Xiao-Jun Wu

**Affiliations:** 1 School of Artificial Intelligence and Computer Science, Jiangnan University, Wuxi 214122, China; dlinzzhang@gmail.com (D.Z.);; 2 Key Laboratory of Intelligent Information Processing, Institute of Computing Technology, Chinese Academy of Sciences, Beijing 100086, China

**Keywords:** food nutrition estimation, RGB-D fusion, ingredient-guided modeling, feature learning

## Abstract

In recent years, food nutrition estimation has received growing attention due to its critical role in dietary analysis and public health. Traditional nutrition assessment methods often rely on manual measurements and expert knowledge, which are time-consuming and not easily scalable. With the advancement of computer vision, RGB-based methods have been proposed, and more recently, RGB-D-based approaches have further improved performance by incorporating depth information to capture spatial cues. While these methods have shown promising results, they still face challenges in complex food scenes, such as limited ability to distinguish visually similar items with different ingredients and insufficient modeling of spatial or semantic relationships. To solve these issues, we propose an Ingredient-Guided Semantic Modeling Network (IGSMNet) for food nutrition estimation. The method introduces an ingredient-guided module that encodes ingredient information using a pre-trained language model and aligns it with visual features via cross-modal attention. At the same time, an internal semantic modeling component is designed to enhance structural understanding through dynamic positional encoding and localized attention, allowing for fine-grained relational reasoning. On the Nutrition5k dataset, our method achieves PMAE values of 12.2% for Calories, 9.4% for Mass, 19.1% for Fat, 18.3% for Carb, and 16.0% for Protein. These results demonstrate that our IGSMNet consistently outperforms existing baselines, validating its effectiveness.

## 1. Introduction

Food nutrition plays a vital role in both daily diet management and clinical nutrition planning, particularly as public attention to health and wellness continues to increase [1]. In its earlier stages, nutrition assessment primarily relied on traditional biochemical methods [2,3]. While these conventional methods are well-established, they often require domain expertise for operation and interpretation, limiting their accessibility and scalability. Furthermore, traditional nutritional assessment methods are often labor-intensive and time-consuming. These limitations hinder their ability to meet the growing demand for rapid, accessible, and precise nutritional evaluation [4,5,6]. To address these issues, recent developments [7,8,9,10,11] in artificial intelligence [12,13,14], particularly in computer vision, have opened new possibilities for automated, accurate, and scalable nutritional assessment, offering a promising alternative to conventional approaches.

In recent years, some vision-based nutritional assessment methods [11,15] have been proposed. These methods employ deep learning [16,17,18,19,20] or machine learning [21,22,23,24] to analyze food images. They learn visual representations that support the direct prediction of nutritional content from visual data. By directly using the visual feature representation, such approaches aim to simplify the assessment process and minimize manual intervention. For instance, Swin-nutrition [25] integrates the Swin Transformer [26] with a feature fusion module and a nutrient prediction module to estimate nutritional content directly from images. Similarly, RoDE [27] employs a mixture-of-experts framework that dynamically adapts to varying task complexities, enhancing precision in nutritional estimation. Despite these advancements, these methods remain constrained by several challenges. For example, fluctuations in lighting conditions can distort the quality of RGB images and subtle food components are often difficult to detect due to their low visual contrast and the limited saliency in RGB images. Moreover, a more fundamental limitation lies in the absence of spatial or depth information, which restricts the ability to capture structural cues from food images. These challenges reduce the performance of nutrient estimation. Therefore, combining depth information with RGB images provides a promising direction to improve the performance of vision-based nutritional estimation methods.

To address the limitations of RGB-only methods, recent research has explored RGB-D-based nutritional assessment, where depth information is integrated with RGB images to provide richer spatial and structural cues. These methods [19,28,29] generally outperform purely RGB-based models. Building on this direction, several representative methods have been developed, including Google-Nutrition [28], IGFNutrition [10], and ADFE [19]. To be specific, Google-Nutrition first investigates depth information for nutrition estimation, showing that spatial cues can significantly enhance nutrient prediction performance. Afterwards, IGFNutrition further combines RGB images, depth maps, and ingredient information to build a more comprehensive food representation for nutritional analysis. ADFE adopts window-based and shifted-window self-attention to enhance visual feature learning, improving the performance siginificantly. While these methods have achieved good results, several challenges remain, such as distinguishing visually similar dishes with different compositions and capturing fine-grained spatial or semantic relationships. Therefore, how to develop a more effective RGB-D-based method that can fully exploit the complementary strengths of visual and depth information remains an open and pressing problem.

To solve the above problem, we propose an Ingredient-Guided Semantic Modeling Network (IGSMNet) for food nutrition estimation. The developed IGSMNet contains two core components: an ingredient-guided module and an internal semantic modeling scheme. Specifically, the first module focuses on bridging ingredient semantics and visual representation. We utilize a dual-branch network to extract multi-scale features from RGB and depth images, capturing complementary texture and spatial cues. The second module addresses the need for internal spatial and contextual reasoning. It comprises two components: the dynamic position encoding and a fine-grained semantic modeling mechanism. The DPE mechanism introduces learnable spatial bias into the attention process, helping the model preserve relative position information. Meanwhile, the fine-grained modeling component enhances local semantic interaction by allowing each feature point to attend to its surrounding context. These components together enrich the internal dependencies among features. The contributions of this work are summarized as follows:

- We propose a novel Ingredient-Guided Semantic Modeling Network (IGSMNet) for food nutrition estimation, which jointly integrates RGB-D visual features and ingredient semantics to enhance the estimation accuracy.
- We develop an ingredient-guided fusion module that utilizes ingredient information to guide visual feature learning. This enables the network to focus on nutritionally relevant regions and enhances its discrimination.
- We introduce an internal semantic modeling strategy composed of dynamic position encoding and fine-grained semantic modeling, which collectively strengthen contextual feature representation.
- Extensive experiments on the Nutrition5k dataset show that the proposed IGSMNet can achieve promising results.

The remainder of this paper is organized as follows: Section 2 briefly reviews the related work. Section 3 introduces the proposed method in detail, and Section 4 reports the experimental results. A discussion is provided in Section 5. Finally, we conclude the work in Section 6.

## 2. Related Work

In the early stages, nutritional assessment primarily relied on manual recording and laboratory-based analysis. While these traditional methods [2,3] are effective, they are often time-consuming and require significant human effort. Recently, with the development of deep learning, a variety of vision-based methods have emerged, enabling faster and more accessible nutritional estimation. In this section, we mainly review recent advances in vision-based nutritional assessment. Current approaches in this field can be broadly categorized into three groups: methods based on RGB images, those incorporating RGB-D data, and methods guided by ingredient information.

### 2.1. RGB Image-Based Methods

Early RGB-based methods mainly relied on convolutional neural networks (CNNs) to extract visual features from food images for nutrition estimation. For example, NR et al. [30] utilize CNNs to learn image features for direct prediction of nutritional content. To enhance the relevance of visual features to caloric estimation, Ege et al. [31] formulate food classification as an auxiliary objective, exploiting the inherent association between food types and caloric content. The approach performs simultaneous dish localization and calorie estimation through a multitask learning scheme. Similarly, Fang et al. [32] observe a strong correlation between energy density and caloric distribution and propose a generative adversarial network to produce energy density maps, improving the relevance of visual cues to calorie estimation. To capture more complex dependencies beyond the capability of CNNs, Shao et al. [25] adopt a transformer-based model and perform multi-scale feature fusion for better representation. In response to the limited performance of direct regression approaches, Wang et al. [33] reformulate the estimation task as classification and design a coarse-to-fine strategy using a structured smoothing objective function. With the increasing application of large multimodal models in vision tasks. Jiao et al. [27] propose a linear rectification mixture of experts to address task conflicts during multi-objective fine-tuning. While these RGB-based methods achieve competitive results, they lack the spatial depth cues necessary for estimating volume, which is critical for accurate nutrient assessment. As a result, recent research has begun to explore the integration of RGB and depth information to address this limitation.

### 2.2. RGB-D Image-Based Methods

Recent works have incorporated depth information to perform the nutrition estimation task, aiming to address the limitations of RGB images in capturing spatial information. For example, Meyers et al. [34] formulate the caloric estimation task as a two-step process, where food class recognition is first used to assign predefined caloric densities and volume is subsequently inferred from depth data to compute the final nutritional value. Based on this, Thames et al. [28] integrate depth images directly into the visual input by appending them as a fourth channel to the RGB representation, which is then processed by a CNN to assess nutritional information, including Calories, Proteins, Fats, and Carbs. Although RGB-D input is effective, early methods often suffer from insufficient modeling of cross-modal relationships and insufficient exploitation of multi-scale features. To solve these limitations, many methods have been developed. For instance, Shao et al. [11] introduce the RGB-D network, which employs multi-scale feature aggregation and cross-modal fusion schemes to capture fine-grained structure, thereby improving estimation performance. Ma et al. [8] develop the FBFPN model, which incorporates a bidirectional feature pyramid alongside an RGB-D fusion module to enhance visual representation. In addition, considering that depth images are difficult to obtain in practical applications, Han et al. [35] propose DPF-Nutrition, which leverages a depth prediction module and fuses the generated depth with RGB features via an attention scheme. Although these RGB-D-based approaches can achieve promising results, they fail to account for ingredient-level information embedded in food images. Therefore, how to incorporate ingredient cues to improve nutritional estimation needs to be further explored.

### 2.3. Ingredient-Guided Methods

To address the issue of RGB-D-based methods in handling visually overlooked ingredient information, recent studies have explored the integration of ingredient-level semantics to further enhance nutritional assessment. For instance, Nian et al. [36] represent ingredient information using textual descriptors, which are encoded into semantic features and aligned with visual representations to enhance representational completeness. Feng et al. [10] perform RGB-D feature fusion by transforming visual representations into the frequency domain and employing a hierarchical multi-scale fusion scheme. Moreover, to enhance semantic alignment, ingredient information is subsequently incorporated as guidance during the feature learning, improving nutritional estimation performance. While existing methods have demonstrated the efficacy of using ingredient information, most existing methods perform ingredient guidance at a coarse level and lack schemes to effectively model the fine-grained contextual dependencies between ingredients and visual features. Therefore, how to combine ingredient information and improve the effectiveness of semantic modeling needs further research.

## 3. Method

In this section, we first provide an overview of our proposed model, followed by a detailed description of its individual modules.

### 3.1. Overview

**Figure 1:**
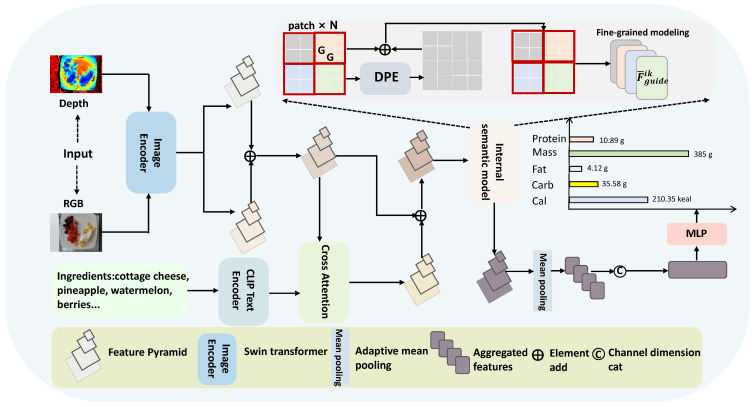
The framework of our IGSMNet, which contains two primary modules. The first is the ingredient-guided module and the second is the internal semantic modeling module.

Figure 1 illustrates the overall structure of our method, which is composed of two primary modules. The first is the ingredient-guided (IG) module, which incorporates the ingredient information to enhance visual representations, enabling the model to better associate visual patterns with nutritional content. The second is the internal semantic modeling (ISM) module, which aims to improve the semantic expressiveness of the visual feature pyramid through intrinsic semantic perception. Moreover, the second module includes two subcomponents: dynamic positional encoding and fine-granularity modeling. The positional encoding dynamically adapts to features at different scales. The fine-granularity modeling component captures contextual relationships among visual elements at each level of the pyramid. Together, these two modules significantly enhance the representational capacity of the constructed food feature pyramid, thereby improving the performance.

In the proposed method, we take as input an RGB image $X_{\mathrm{rgb}}\in R^{H\times W\times 3}$ and its corresponding depth image $X_{\mathrm{depth}}\in R^{H\times W\times 3}$, where *H* and *W*, respectively, denote the image height and width. To extract modality-specific features, an asymmetric dual-branch architecture is employed, with each branch structured as a four-stage hierarchical network. The spatial resolution is progressively reduced by half at each stage, thereby increasing the receptive field and enhancing semantic abstraction. This process yields multi-scale feature sets for both modalities, represented as $F_{\mathrm{rgb}}=\left\{F_{\mathrm{rgb}}^{i}|i=1,\;2,\;3,\;4\right\}$ and $F_{\mathrm{depth}}=\left\{F_{\mathrm{depth}}^{i}|i=1,\;2,\;3,\;4\right\}$. Meanwhile, high-level features are further enriched through semantic fusion with CLIP-derived embeddings, while the ingredient-guided and internal semantic modules jointly enhance representation learning. Finally, a multitask learning framework is employed to infer the content of five nutritional ingredients.

### 3.2. Ingredient-Guided Module

In food nutrition estimation, it is essential to not only recognize ingredients based on visual appearance but also to understand their spatial information, as this directly impacts the accuracy of portion and nutrient assessment. While RGB images capture rich visual cues such as color and surface texture, they lack the ability to describe the spatial information (such as depth and volume). To address this issue, the depth images are leveraged in this work, which can help the model estimate size and spatial arrangement more precisely. To fully exploit the complementary strengths of RGB and depth modalities, we utilize a hierarchical fusion scheme. Specifically, we integrate features from both modalities at different levels. The formulation can be defined as follows:

(1) $$F_{\mathrm{fused}}^{i}=F_{\mathrm{rgb}}^{i}+F_{\mathrm{depth}}^{i},$$

where $F_{\mathrm{fused}}^{i}\in R^{B\times C\times H\times W}$ represents the fused feature at the *i*-th level. By integrating features from both RGB and depth modalities across scales, it can concurrently exploit appearance-based information and spatial structure. Moreover, lower-level representations can preserve detailed spatial and edge information, while higher-level features encode abstract semantic relationships.

**Figure 2:**
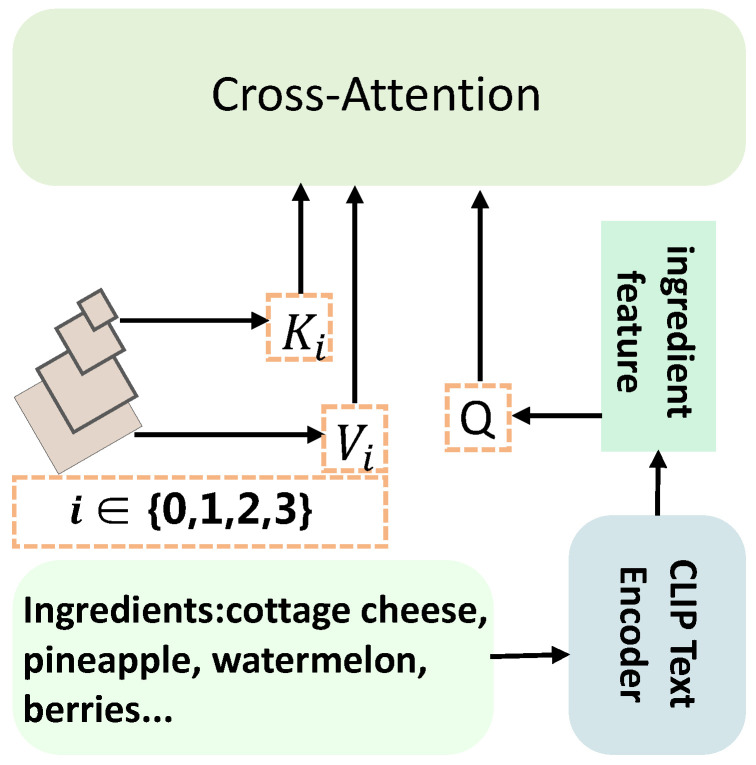
The framework of our ingredient-guided module.

To further improve the accuracy of food nutrition estimation, we introduce ingredient knowledge as a semantic guide for visual feature learning. By embedding ingredient information into the model, the network can be guided to attend to visual regions that are semantically aligned with specific food components, thereby improving its ability to recognize individual ingredients. As a result, the guidance mechanism directly contributes to more accurate and reliable nutritional estimation. Specifically, we utilize the pre-trained CLIP textual encoder to encode the ingredient information, which can be defined as follows:

(2) $$F_{\mathrm{ingredient}}=\mathrm{CLIP}\left(\mathrm{ingredients}\right),$$

where $F_{\mathrm{ingredient}}\in R^{B\times C}$ represents the food ingredient features, *B* is the batch size, and *C* is the embedding dimensionality. To facilitate the guidance of visual feature extraction using ingredient semantics, we use a cross-attention scheme [16] that captures the interaction between ingredient semantics and visual information. Prior to attention computation, we convert the two features into a compatible shape to allow effective alignment: $F_{\mathrm{ingredient}}\in R^{B\times 1\times C}$ and $F_{\mathrm{fused}}^{i}\in R^{B\times (H\times W)\times C}$. Specifically, as illustrated in Figure 2, the cross-attention strategy is used to associate textual ingredient features with visual representations. The ingredient embedding serves as the query, while the fused visual features act as both keys and values. Then, we have:

(3) $$Q=W_{Q}F_{\mathrm{ingredient}},\;K=W_{K}F_{\mathrm{fused}}^{i},\;V=W_{V}F_{\mathrm{fused}}^{i},$$

where $W_{Q},W_{K},W_{V}\in R^{C\times C}$ are linear transformation matrices for the query, key, and value, respectively. The output of cross-attention is computed as:

(4) $$\mathrm{CrossAttention}\left(Q,\;K,\;V\right)=\mathrm{softmax}\left(\frac{QK^{T}}{\sqrt{d_{k}}}\right)V,$$

where $d_{k}$ denotes the dimension of the keys, which in this case equals *C*. Combining the above elements, we obtain the ingredient-guided visual feature at each scale:

(5) $$F_{\mathrm{guide}}^{i}=\mathrm{softmax}\left(\frac{\left(W_{Q}F_{\mathrm{ingredient}}\right){\left(W_{K}F_{\mathrm{fused}}^{i}\right)}^{T}}{\sqrt{C}}\right)\left(W_{V}F_{\mathrm{fused}}^{i}\right).$$

The above operations yield the ingredient-guided feature map $F_{\mathrm{guide}}^{i}$ at scale *i*, in which the visual representation is enhanced based on the semantic relevance of the corresponding ingredient information. To ensure that the original visual cues are preserved during this guidance process, we incorporate a residual connection by summing the guided feature with its corresponding fused visual representation:

(6) $${\bar{F}}_{\mathrm{guide}}^{i}=F_{\mathrm{guide}}^{i}+F_{\mathrm{fused}}^{i}.$$

### 3.3. Internal Semantic Modeling

**Figure 3:**
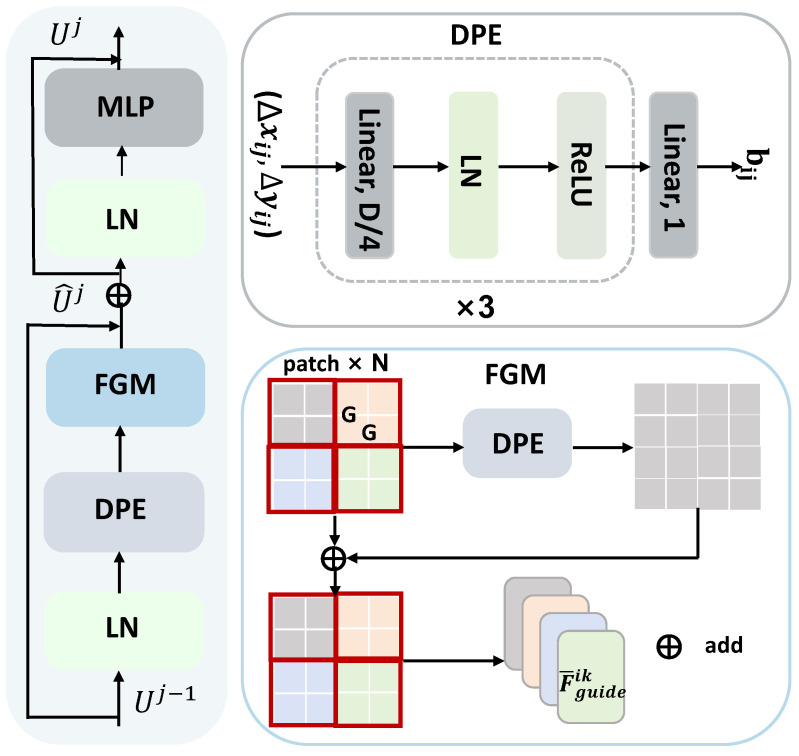
The framework of the internal semantic modeling scheme.

After obtaining the ingredient-guided features, we observe that while these representations incorporate ingredient semantic information, they lack explicit modeling of internal spatial and compositional relationships among food components. To this end, and inspired by [37,38], we develop the internal semantic modeling strategy to further enhance representational capacity. Figure 3 shows the framework. This module can capture structural dependencies among feature elements by modeling the internal relationships within each feature map. Specifically, the proposed module consists of two key components. First, dynamic positional encoding is applied to introduce position-specific representations, enabling the network to differentiate visual elements based on their spatial locations. Second, fine-grained semantic modeling is used to capture local contextual relationships, ensuring that each feature element can attend to its surrounding semantic structure.

#### 3.3.1. Dynamic Position Encoding

To encode relative spatial information among features, we adopt a dynamic position encoding (DPE) mechanism, which introduces learnable positional bias into the attention computation. Specifically, the attention map in the internal semantic modeling (ISM) module is rewritten as follows:

(7) $$\mathrm{Attnmap}=\mathrm{Softmax}(QK^{T}/\sqrt{d}+b)V,$$

where $Q,\;K,\;V\in R^{G^{2}\times D}$ denote the query, key, and value matrices, respectively, and *d* is a scaling factor for normalization, D is the embedding dimension, and G is the size of patch and will be formally defined in the next section. The term $b\in R^{G^{2}\times G^{2}}$ represents the relative position bias matrix introduced by DPE. In [26], the bias is computed by a fixed table $b_{i,j}={\hat{b}}_{\Delta x_{ij},\Delta y_{ij}}$ where $\hat{b}$ is a fixed-sized matrix and $\left(\Delta x_{ij},\;\Delta y_{ij}\right)$ is the offset between tokens *i* and *j*. However, this scheme lacks adaptability to varying patch sizes across feature pyramid levels. To address this limitation, we introduce a dynamically generated relative position module named DPE, which is defined as:

(8) $$b_{i,j}=\mathrm{DPE}\left(\Delta x_{ij},\Delta y_{ij}\right),$$

where DPE is implemented as a multi-layer perceptron (MLP). It takes the offset $\left(\Delta x_{ij},\Delta y_{ij}\right)$ as input and produces a scalar output. The scheme consists of three fully connected layers with ReLU activations [39] and LayerNorm [40], and the intermediate layer is set to dimension D/4. This design ensures the learned positional bias can adapt to different spatial contexts and generalize across variable input resolutions.

#### 3.3.2. Fine-Grained Modeling

To further capture local dependencies within patches, we employ a fine-grained modeling (FGM) mechanism. Given an ingredient-guided feature map ${\bar{F}}_{\mathrm{guide}}^{i}$ at level *i*, we partition it into *N* non-overlapping square patches of size G×G. Each patch is independently flattened and projected into queries, keys, and values. Then, *Q*, *K*, and *V* are $f_{Q}={\bar{F}}_{\mathrm{guide}}^{ik}W^{Q}$, $f_{K}={\bar{F}}_{\mathrm{guide}}^{ik}W^{K}$, and $f_{V}={\bar{F}}_{\mathrm{guide}}^{ik}W^{V}$, respectively. For each attention head *h* in a multi-head self-attention module with *H* heads, the attention weights are computed as:

(9) $$W_{1}^{h}=\mathrm{softmax}\left(\frac{f_{Q1_{h},i}f_{K1_{h},j}^{T}}{\sqrt{d/H}}\right),$$

where D/H is the feature dimension processed by each head. For FGM, *k* and *j* denote the index of the embedding, and *h* is the index of the head. The output for all heads is aggregated as:

(10) $$f_{FGM}=\sum_{h=1}^{H}\sum_{k,j=1}^{G}W_{1}^{h}\cdot f_{V1_{h,\;j}},$$

In addition, the internal semantic modeling strategy contains four components: DPE, FGM, a normalization layer (LayerNorm, LN), and a multilayer perceptron (MLP). The FGM module adopts a window-based multi-head self-attention scheme, where attention blocks are alternated and each block is equipped with residual connections. The transformer block applied to image input $x_{i}$ can be defined as:

(11) $$\begin{array}{cc} {\hat{U}}^{j} & =\mathrm{FGM}\left(\mathrm{LN}(U^{j-1})\right)+U^{j-1},\\ U^{j} & =\mathrm{MLP}\left(\mathrm{LN}({\hat{U}}^{j})\right)+{\hat{U}}^{j}, \end{array}$$

where $U^{j-1}$ is the input from the previous block and LN is the LayerNorm operation. The initial input ${\bar{F}}_{\mathrm{guide}}^{i}$ passes through *L* stacked ISM blocks, yielding the final refined feature representation at each pyramid level, denoted by $F_{ism}^{i}$.

### 3.4. Training Objective

After obtaining the final feature pyramid $F_{\mathrm{ism}}$, we perform global feature aggregation to facilitate nutritional prediction. Specifically, each level of the pyramid is processed using adaptive average pooling. Then, we have:

(12) $$P_{i}=\mathrm{AdaptiveAvgPool}2d\left(F_{\mathrm{ism}}^{i},\;\text{output\_size}=\left(1,1\right)\right),$$

The pooled outputs from all four pyramid levels are then concatenated along the channel dimension:

(13) $$F_{\mathrm{concat}}=Cat\left(\left(P_{1},P_{2},P_{3},P_{4}\right),dim=-1\right),$$

where $F_{\mathrm{concat}}\in B\times \left(C\times 4\right)$. Then, the concatenated vector is then passed through a series MLP layers to obtain the corresponding predicted results. Specifically, our method is trained using a multitask learning scheme to jointly predict five nutrients (i.e., Calories, Mass, Fats, Proteins, and Carbohydrates). As the value scales of these nutrients vary considerably, we normalize the loss measures for each nutritional ingredient and apply the loss function as follows:

(14) $$L=l_{\mathrm{cal}}+l_{\mathrm{carb}}+l_{\mathrm{pro}}+l_{\mathrm{fat}}+l_{\mathrm{mass}},$$

where $l_{\mathrm{cal}},l_{\mathrm{mass}},l_{\mathrm{pro}},l_{\mathrm{carb}},$ and $l_{\mathrm{fat}}$ represent the loss for Calories, Mass, Proteins, Carbohydrates, and Fats, respectively. each component is computed as the normalized absolute error between the predicted and ground-truth values. For example, the result of $l_{\mathrm{cal}}$ is:

(15) $$l_{\mathrm{cal}}=\frac{\sum_{i=1}^{N}\left|y_{i}-{\hat{y}}_{i}\right|}{\sum_{i=1}^{N}y_{i}},$$

where $y_{i}$ is the true nutritional value and ${\hat{y}}_{i}$ is the predicted nutritional value. The same formulation is applied to all five tasks, ensuring consistent error metrics across different nutrient types.

### 3.5. Evaluation Metrics

To quantitatively assess the efficacy of our method, we adopt the percentage of mean absolute error (PMAE) as the metric. Let N denote the number of samples. The mean absolute error (MAE) is computed as:

(16) $$\mathrm{MAE}=\frac{1}{N}\sum_{i=1}^{N}\left|y_{i}-{\hat{y}}_{i}\right|.$$

Based on this, PMAE is defined by normalizing MAE. Then, PMAE can be defined as:

(17) $$\mathrm{PMAE}=\frac{\mathrm{MAE}}{\frac{1}{N}\sum_{i=1}^{N}y_{i}},$$

where PAME reflects the prediction error relative to the scale of true values. A smaller PMAE indicates better estimation accuracy.

## 4. Experiments

### 4.1. Experimental Setup

We conduct experiments on the Nutrition5k [28] dataset. Specifically, the dataset contains 5000 dishes across a wide range of categories and includes annotations for over 250 types of ingredients. The dataset provides single-view and $360^{{}^{\circ}}$ captures, from which we select top-view RGB-D pairs acquired using an Intel RealSense D435 camera [28]. The data is partitioned into training and test sets following a 5:1 ratio. More detailed information can be found referring to [28]. All experiments are implemented on a workstation equipped with an NVIDIA GTX 3090 GPU. The backbone network is initialized with ImageNet-pre-trained weights. Input images are uniformly cropped and resized to 224×224. Moreover, we apply synchronous inversion augmentation to the RGB-D training samples to improve the robustness. Model optimization is carried out using the Adam optimization algorithm, starting with a learning rate of $5\times 10^{-5}$ that decays exponentially at a rate of 0.99. Training is performed for 150 epochs with a batch size of 32.

### 4.2. Experimental Results and Analysis

In this section, we conduct a comprehensive comparison between our IGSMNet and some recent competitive methods on the Nutrition5k dataset. To the best of our knowledge, The compared approaches include representative RGB-based methods, such as Google-Nutrition-rgb [28], Portion-Nutrition [41], Swin-nutrition [25], and DPF-Nutrition [35]. In addition, we evaluate against recent RGB-D fusion approaches, including CMX [42], HINet [29], CDINet [43], DEFNet [44], TriTransNet [26], Deliver [45], Google-Nutrition-rgbd [28], IMIR-Net [36], and Feng et al. [10]. The experimental results are given in Table 1.

#### 4.2.1. Comparison with RGB Image-Based Methods

As shown in Table 1, we can see that the proposed IGSMNet outperforms all RGB-only baselines. Our IGSMNet achieves consistent improvements across all tasks. Specifically, in terms of the overall average, our model obtains a PMAE of 15.0%, surpassing the best-performing RGB-based method, DPF-Nutrition [35], which reports 17.8%. Regarding specific nutritional estimation tasks, our method also demonstrates clear advantages. For instance, the PMAE for Protein estimation is reduced from 20.2% to 16.0%, marking a 4.2% improvement. On the Mass metric, our approach yields a PMAE of 9.4%, outperforming the 10.6% reported by DPF-Nutrition by 1.2%. These results demonstrate that our framework, by incorporating richer information beyond RGB inputs, significantly enhances prediction accuracy. Overall, the proposed method consistently outperforms RGB-based approaches, highlighting its effectiveness in addressing the limitations of RGB-only nutritional estimation.

#### 4.2.2. Comparison with RGB-D-Based Methods

We also compare with RGB-D-based methods, as summarized in Table 1. It can be observed that our proposed approach achieves consistent improvements over existing RGB-D fusion approaches in predicting most nutritional components. Specifically, our method achieves a mean PMAE of 15.0%, significantly outperforming all other methods. For instance, the best-performing compared approach, Google-Nutrition-rgbd, reports a mean PMAE of 20.1%, indicating a substantial 5.1% improvement obtained by our model. In terms of individual nutrients, our model consistently reports the lowest PMAE values: 12.2% for Calories, 9.4% for Mass, 19.1% for Fat, 18.3% for Carb, and 16.0% for Protein. Compared to these RGB-D-based methods such as Google-Nutrition-rgbd and Deliver, which rely on cross-modal attention or multimodal feature alignment schemes, our model exhibits a clear advantage in prediction accuracy. Moreover, the Mass metric shows limited improvement because it depends mainly on physical cues such as food volume and thermal radiation, which are not the primary focus of the IG and ISM modules. These modules are designed to enhance ingredient-level semantics and contextual reasoning, leading to clear gains in nutrient estimation but only marginal benefits for Mass prediction. It is worth noting that while most methods suffer from high PMAE in Fat and Protein prediction, our model maintains relatively low errors, indicating better generalization across nutrients. The better performance of our method can be attributed to two main factors: (1) the ingredient-guided scheme, which provides explicit semantic information aligned with nutrient properties, and (2) the fine-grained semantic modeling module, which further promotes the integration and complementarity between visual representations and ingredient information.

### 4.3. Ablation Study

This section analyzes the impact of different components on nutritional assessment performance. Specifically, we conduct ablation studies on the ingredient-guided scheme, the fine-grained modeling strategy, and the dynamic positional encoding module. The experimental results and a detailed analysis are presented below.

#### 4.3.1. Effectiveness of the Ingredient-Guided Module

As shown in Table 2, the ingredient-guided module plays a critical role in enhancing estimation performance. When this module is introduced into the baseline model, the overall prediction error (mean PMAE) is reduced from 16.9% to 15.6%, yielding a 1.3% improvement. For the estimation of the individual nutrient predictions, all components except Mass benefit from this module. In particular, the PMAE for Calories drops from 13.7% to 13.3%, Fat from 22.6% to 19.5%, Carb from 19.4% to 18.5%, and Protein from 19.6% to 16.6%. These results indicate that ingredient-level information contributes significantly to the semantic alignment between visual features and nutrition-related properties. Although the ingredient-guided module effectively improves predictions for most nutritional components, a slight performance drop is observed for the Mass. This may be due to the weak correlation between ingredient semantics and physical quantity, as ingredient presence does not directly reflect the Mass.

#### 4.3.2. Effectiveness of the Fine-Grained Modeling Scheme

As shown in the fourth row of Table 2, incorporating the FGM scheme leads to a further reduction in mean PMAE, from 15.6% to 15.2%, representing a 0.4% improvement. This gain is primarily reflected in the predictions of Calories, Mass, and Protein. Compared to using the ingredient-guided module alone, where the Mass error increases, the inclusion of FGM reduces the Mass PMAE from 10.2% to 9.4%. This indicates that fine-grained modeling strengthens the internal structure of visual features and effectively preserves semantics unrelated to ingredient information. In addition, the improved modeling of fine-grained information contributes to a more stable and discriminative representation, enhancing the effectiveness of ingredient-guided features.

#### 4.3.3. Effectiveness of the Dynamic Position Encoding

As observed in the last row of Table 2, introducing the DPE module leads to a further reduction in mean PMAE from 15.2% to 15.0%, indicating a marginal improvement of 0.2%. This improvement is primarily reflected in the predictions of Calories, Fat, and Protein. The performance gain indicates that, without position encoding, the model may not capture spatial associations among visual elements, limiting its ability to identify whether features belong to the same food item. By incorporating DPE, the model can establish positional context across local regions, reinforcing the semantic coherence of visual features and improving the performance.

### 4.4. Further Analysis

#### 4.4.1. Comparison of Ingredient-Guided Integration Strategies

To explore the efficacy of different ingredient-guided integration strategies, we conduct some experiments (including Add, MLP, and Cross-Attention schemes); the results are given in Table 3. It can be observed that the incorporation of ingredient-guided schemes, regardless of the specific integration strategy, leads to consistent performance improvements compared to the baseline without ingredient guidance. Among these schemes, the cross-attention strategy achieves the best result, reducing the mean PMAE to 15.0% and yielding the lowest errors for Calories, Fat, Carb, and Protein. In contrast, the Add and MLP strategies yield less substantial improvements, with mean PMAE values of 16.5% and 16.6%, respectively. The performance advantage of the cross-attention scheme indicates its efficacy in capturing the relationships between ingredient information and visual features. Compared with the Add and MLP strategies, the cross-attention scheme effectively establishes associations between ingredient information and relevant visual regions. This interaction suppresses interference from unrelated ingredients and improves the semantic relevance of the fused features. As a result, the cross-attention scheme achieves more accurate overall predictions and enhances the precision of nutritional component estimation.

#### 4.4.2. Modeling Order of IG and ISM

As shown in Table 4, we can see that incorporating the ISM strategy improves overall performance, regardless of the integration order. Specifically, both modeling orders outperform the single IG baseline, with mean PMAEs reduced from 15.6% to 15.0% and 15.1%, respectively. Notably, applying IG before ISM achieves slightly better results across most nutritional components. The improvement is mainly due to the fact that IG introduces semantic alignment but may weaken local visual details. Using ISM after IG helps recover this lost information by modeling contextual dependencies, thus improving overall performance. In contrast, when ISM is applied first, this refinement cannot occur, leading to reduced performance in certain components, such as Carb. These results indicate that the order of applying IG before ISM is more effective for maintaining semantic consistency and improving prediction accuracy.

## 5. Discussion

Vision-based nutritional analysis offers an efficient and scalable solution for estimating nutrient composition directly from food images, supporting individualized dietary monitoring and nutritional intervention. Although our method achieves good results compared to existing baselines, several limitations are observed. Specifically, the proposed method shows no improvement on the Mass task. This may result from the weak association between ingredient information and physical quantity, as ingredient presence does not directly reflect volume or density. Consequently, the model may lack sensitivity to structural information. Moreover, while the model leverages ingredient information as a guiding signal, it assumes complete and accurate information availability. In practical scenarios, ingredient annotations are often partial, noisy, or user-provided. The current model may lack robustness under such conditions. This highlights the need for more flexible schemes that can adapt to uncertain or partial ingredient descriptions while maintaining promising performance. In addition, another promising direction is the integration of personalization into nutritional assessment, where user history and dietary patterns are incorporated to achieve more practical outcomes.

## 6. Conclusions

In this paper, we propose a novel food nutrition estimation method named IGSMNet, which integrates ingredient information with RGB-D visual features to further improve the performance. The proposed method first combines RGB images with depth information to extract semantically enriched RGB-D representations. Ingredient information is then employed to guide these representations, enhancing their alignment with nutritional attributes. To further refine feature discrimination, an internal semantic modeling scheme is introduced, which performs fine-granularity encoding to capture local contextual dependencies among fused features. In addition, a dynamic position encoding module is incorporated to strengthen the ability to perceive spatial relations. Experimental results on the Nutrition5k dataset demonstrate the superior performance of the developed IGSMNet compared with existing baselines. In the future, we will explore lightweight model designs to improve efficiency and facilitate deployment in real-world nutritional assessment scenarios.

## Figures and Tables

**Table 1:** Performance comparison of our IGSMNet and other methods on Nutrition5K; the best results are highlighted in bold.

Method Type	Methods	PMAE (%)					
		Calories	Mass	Fat	Carb	Protein	Mean
RGB images	Google-Nutrition-rgb [28]	26.1	18.8	34.2	31.9	29.5	29.1
	Coarse-to-Fine Nutrition [33]	24.1	19.4	36.0	32.1	33.5	29.0
	Swin-nutrition [25]	16.2	13.7	24.9	21.8	25.4	20.4
	Portion-Nutrition [41]	15.8	-	-	-	-	-
	RoDE [27]	52.4	38.4	67.1	47.8	53.9	51.9
	DPF-Nutrition [35]	14.7	10.6	22.6	20.7	20.2	17.8
RGB-D images	CMX [42]	21.8	20.7	34.8	37.0	33.2	29.5
	HINet [29]	24.5	25.2	43.4	39.9	38.8	34.3
	CDINet [43]	21.1	20.4	37.1	37.1	32.8	29.7
	DEFNet [44]	32.7	34.2	48.9	40.3	43.8	39.9
	TriTransNet [26]	22.1	20.1	37.5	34.8	38.0	30.5
	Deliver [45]	29.5	25.9	48.3	47.7	46.1	39.5
	Google-Nutrition-rgbd [28]	18.8	18.9	**18.1**	23.8	20.9	20.1
	IMIR-Net [36]	14.7	11.4	23.3	20.9	21.6	18.4
	Feng et al. [10]	13.7	9.8	19.2	19.3	17.6	15.9
	IGSMNet	**12.2**	**9.4**	19.1	**18.3**	**16.0**	**15.0**

**Table 2:** Ablation study evaluating the impact of each module (the best results are marked in bold).

Baseline	IG	FGM	DPE	Calories	Mass	Fat	Carb	Protein	Mean
**✓**				13.7	9.4	22.6	19.4	19.6	16.9
**✓**	**✓**			13.3	10.2	19.5	18.5	16.6	15.6
**✓**	**✓**	**✓**		12.6	9.4	19.5	18.3	16.2	15.2
**✓**	**✓**	**✓**	**✓**	**12.2**	**9.4**	**19.1**	**18.3**	**16.0**	**15.0**

**Table 3:** Results of different ingredient-guided integration strategies (we mark the best results in bold).

IG Integration Strategy	Calories	Mass	Fat	Carb	Protein	Mean
w/o IG	13.7	9.4	22.6	19.4	19.6	16.9
Add	13.5	9.7	21.8	19.2	18.5	16.5
MLP	13.4	9.3	22.0	19.8	18.8	16.6
Cross-Attention	**12.2**	**9.4**	**19.1**	**18.3**	**16.0**	**15.0**

**Table 4:** Effects of different integration orders between IG and ISM (the best results are marked in bold).

Configuration	Calories	Mass	Fat	Carb	Protein	Mean
w/o IG	13.7	9.4	22.6	19.4	19.6	16.9
IG only	13.3	10.2	19.5	18.5	16.6	15.6
ISM → IG	12.9	9.7	**18.3**	19.1	**15.5**	15.1
IG → ISM	**12.2**	**9.4**	19.1	**18.3**	16.0	**15.0**

## Data Availability

The data is available at https://github.com/dmcsy/ISMIG, accessed on 28 September 2025.
